# Chemokine (C-C motif) ligand 2 mediates direct and indirect fibrotic responses in human and murine cultured fibrocytes

**DOI:** 10.1186/1755-1536-4-23

**Published:** 2011-10-19

**Authors:** Jason E Ekert, Lynne A Murray, Anuk M Das, Hai Sheng, Jill Giles-Komar, Michael A Rycyzyn

**Affiliations:** 1 Departments of Cell Biology and Assay Technologies, Centocor R&D, a division of Johnson & Johnson Pharmaceutical Research & Development, LLC, Radnor, PA, USA; 2 Department of Immunology, Centocor R&D, a division of Johnson & Johnson Pharmaceutical Research & Development, LLC, Radnor, PA, USA

## Abstract

**Background:**

Fibrocytes are a population of circulating bone-marrow-derived cells that express surface markers for leukocytes and mesenchymal cells, and are capable of differentiating into myofibroblasts. They have been observed at sites of active fibrosis and increased circulating numbers correlate with mortality in idiopathic pulmonary fibrosis (IPF). Inhibition of chemokine (C-C motif) receptor 2 (CCR2) during experimental models of lung fibrosis reduces lung collagen deposition, as well as reducing lung fibrocyte accumulation. The aim of the present study was to determine whether human and mouse fibrocytes express functional CCR2.

**Results:**

Following optimized and identical human and murine fibrocyte isolation, both cell sources were shown to be positive for CCR2 by flow cytometry and this expression colocalized with collagen I and CD45. Human blood fibrocytes stimulated with the CCR2 ligand chemokine (C-C motif) ligand 2 (CCL2), demonstrated increased proliferation (*P *< 0.005) and differentiation into myofibroblasts (*P *< 0.001), as well as a chemotactic response (*P *< 0.05). Murine fibrocytes also responded to CCR2 stimulation, with CCL12 being more potent than CCL2.

**Conclusions:**

This study directly compares the functional responses of human and murine fibrocytes to CCR2 ligands, and following comparable isolation techniques. We have shown comparable biological effects, strengthening the translatability of the murine models to human disease with respect to targeting the CCR2 axis to ameliorate disease in IPF patients.

## Background

Fibrocytes are a population of circulating cells that have been reported to express a variety of markers including leukocyte markers (CD45, CD34), mesenchymal markers (collagen I, fibronectin) and chemokine receptors (chemokine (C-C motif) receptor 3 (CCR3), CCR5, CCR7 and C-X-C chemokine receptor type 4 (CXCR4)) [1]. Human and mouse studies have demonstrated that fibrocytes from peripheral blood migrate to skin wound chambers [1-3] and bronchial mucosa after antigen challenge [4]. Furthermore, these cells have been reported in disease states with fibrotic pathologies including hypertrophic scars, asthma and idiopathic pulmonary fibrosis (IPF) [4-7]. Fibrocytes are functionally pleiotropic, potentially contributing to fibrogenesis by directly producing collagen, as well as inflammatory cytokines, hematopoietic growth factors, and chemokines [6-10].

In preclinical models of lung fibrosis, inhibition of various chemokine receptor/ligand pathways reduces lung fibrosis and it has been hypothesized to be in part due to a reduction in fibrocyte recruitment [6-9]. However, the effect of inhibiting specific chemokine receptors expressed on fibrocytes in the clinical setting remains to be elucidated. CCR2 is the high-affinity receptor for its ligand chemokine (C-C motif) ligand 2 (CCL2) (monocyte chemoattractant protein 1 (MCP-1)). We have recently shown that IPF patients have increased CCL2 in the circulation [11]. However, the expression of CCR2 on human fibrocytes has yet to be described. In murine models of fibrosis, CD45^+^ lung fibrocytes isolated from the lung express CCR2 [6]. Inhibition of this chemokine axis through monoclonal antibody neutralization of the ligands resulted in a decrease in fluorescein isothiocyanate (FITC)-induced fibrosis, with a concomitant reduction in fibrocyte number in the lung [7]. Further, *in vitro*, mCCL2/JE and mCCL12/MCP5 promote lung-derived fibrocyte migration [6,7].

Several studies have recently demonstrated increased fibrocytes in the circulation of patients with fibrotic lung disease [12,13], and the presence and modulation of fibrocytes in experimental models of lung fibrosis [6,9,14]. However, one confounding issue when comparing interpretations across different laboratories is that the isolation techniques, fibrocyte cell markers and isolation parameters vary. Therefore, we sought to directly compare fibrocytes from human and mouse blood using an optimized technique, and measured functional responses in the same assay systems, thus enabling cross-species comparisons.

## Methods

### Fibrocyte isolation from human and murine peripheral blood

For collection of human blood, all necessary permissions, approvals, and licenses, including approval by a relevant third party Institutional Review Board (IRB) of an Informed Consent Form and Protocol and any relevant study-related documentation required by the IRB, were collected and maintained for the sourcing, handling, storage, banking, transport, or use of biological samples from employee volunteers at Centocor, R&D, Inc. For BALB/c mouse blood collection, all animal studies were approved by the Centocor Institutional Animal Care and Use Committee (IACUC) in accordance with Federal guidelines. Total peripheral blood mononuclear cells (PBMCs) were isolated from human (60-100 ml of blood was drawn from healthy donors 20-60 years of age) or murine blood (approximately 1 ml of blood was drawn from each mouse (6-10 weeks of age) and the blood was pooled from 10 mice) by centrifugation over Ficoll-Paque (Pharmacia, Uppsala, Sweden) following the manufacturer's protocol. After 2 days in culture on uncoated culture dishes in Dulbecco's modified Eagle medium (DMEM; Life Technologies, Gaithersburg, MD, USA) supplemented with 20% fetal calf serum (FCS; HyClone Labs, Logan, UT, USA), the non-adherent cells were removed by a single, gentle aspiration. Following 10 to 12 days of continuous culture for human blood and 14-18 days for murine blood, the adherent cells were lifted by incubation in Accutase (Millipore, Billerica, MA, USA) and were depleted by immunomagnetic selection of contaminating T cells (pan-T, anti-CD2; Miltenyi Biotech, Auburn, CA, USA), monocytes (anti-CD14, Miltenyi Biotech), and B cells (Pan-B, anti-CD19, Miltenyi Biotech). Cell viability was determined to be >90% by Guava analysis (Millipore). Immunohistochemistry (IHC) and flow cytometry staining of this population confirmed that these cells were positive for both CD45 and collagen-I fibrocyte markers. Typically, the isolated blood fibrocytes from both human (n = 4) and mouse blood (n = 3, 10 mice pooled per experiment) represented less than 0.5% of the starting PBMC pool.

### Immunohistochemistry

For immunofluorescence, cells were seeded into eight-well chamber slides at 1.5 × 10^4^ viable cells/well and were fixed when semiconfluent (day 7) in 3% formaldehyde for 10 min at room temperature and subsequently washed several times in phosphate-buffered saline (PBS). The cells were permeablized with methanol for 10 min at -20°C and washed in staining buffer (0.2% bovine serum albumin (BSA) (Sigma Aldrich, St. Louis, MO, USA) and 0.02% sodium azide in PBS). Blocking of non-specific antibody binding was performed with Universal power blocking solution (Biogenex, San Ramon, CA, USA) for 5 min at room temperature and washed in staining buffer. Blocked slides were incubated for 2 h at room temperature in staining buffer with primary antibodies. Human cells were stained with chicken anti-human CCR2 polyclonal antibody (Genway Biotech Inc, San Diego, CA, USA) and mouse anti-human CD45 monoclonal antibody (eBiosciences, San Diego, CA, USA). Murine cells were stained with chicken anti-human CCR2 polyclonal antibody (Genway Biotech), rat anti-mouse CD45 monoclonal antibody (BD Biosciences, San Jose, CA, USA) and rabbit anti-mouse collagen I polyclonal antibody (Chemicon, Temecula, CA, USA). Murine cells were costained with either CD45 and collagen I, or CCR2 and CD45, or CCR2 and collagen I. Isotype controls were used for non-specific background which included: chicken IgY isotype control (SouthernBiotech, Birmingham, AL, USA), mouse IgG_1_ isotype control (BD Biosciences), rat IgG_2b_ isotype control (BD Biosciences) and rabbit IgG isotype control (Imgenex, San Diego, CA, USA). The cells were washed three times in staining buffer. The secondary antibody was added and incubated for 1 h in the dark. The secondary antibody for CCR2 was goat anti-chicken IgY-FITC (Genway Biotech), CD45 was Alexa Fluor 594 goat anti-rat IgG (H+L) (Molecular Probes, Eugene, OR, USA) and collagen I was Alexa Fluor 647 goat anti-rabbit Ig (H+L) (Molecular Probes). Stained slides were washed three times and mounted (Slowfade Gold antifade with 4',6-diamidino-2-phenylindole (DAPI); Molecular Probes). All slides were viewed on the laser confocal microscope (UltraVIEW ERS spinning disk; Perkin Elmer, Wellesley, MA, USA).

### Flow cytometry analysis

For flow cytometry analysis, 2 × 10^5^ viable cells were added to flow tubes and centrifuged. The cells were resuspended in 50 μl of staining buffer (0.2% BSA and 0.02% sodium azide in PBS) and 1 μl of Fc blocker (BD Biosciences). The cells were incubated for 10 min. Species-relevant phycoerythrin (PE)-conjugated anti- CD45 monoclonal antibodies (BD Biosciences) were added and incubated for 40 min after which the cells were washed three times with staining buffer, fixed and permeabilized by adding BD cytofix/cytoperm (BD Biosciences) for 20 min. The cells were centrifuged and resuspended in blocking solution (BD buffer solution) for 5 min. The cells were incubated for 40 min with chicken anti-human CCR2 primary antibody (Genway Biotech) and biotin anti-collagen I (Rockland, Gilbertsville, PA, USA). The cells were washed three times with BD buffer and the secondary antibody for CCR2, FITC-IgY antibody (Genway Biotech) and for collagen I, streptavidin-APC (BD Biosciences) was added and incubated for 30 min. The cells were washed three times and fixed with 3% paraformaldhyde. Isotype controls used for non-specific background included: chicken IgY isotype control (SouthernBiotech), PErat IgG_2b_ isotype control (BD Biosciences), rat IgG_2a_ isotype control (BD Biosciences), and IgG fraction of anti-Biotin (Rockland).

### Cell proliferation assay

Selected human and murine blood fibrocytes were plated at 0.5 × 10^4^ viable cells/well in a 96 well tissue culture plate. The cells were incubated in control media (10% BSA in DMEM) overnight. The media was removed and replaced with mCCL2 (1 μM equivalent to 8.8 ng/ml) (Peprotech, Rocky Hill, NJ, USA), CCL12 (1 μM equivalent to 9.3 ng/ml) (Peprotech) or platelet-derived growth factor (PDGF)-AB (Peprotech) for murine fibrocytes and hCCL2 (Peprotech) or PDGF-AB for human fibrocytes in serum free media (1% BSA and insulin transferrin selenium (ITS; Invitrogen, Carlsbad, CA, USA) in DMEM). The fibrocyte cells were incubated for 96 h at 37°C in a humidified chamber. The proliferative activity was measured using CellTiter-Glo Luminescent Cell Viability Assay (Promega, Madison, WI, USA). CellTiter-Glo is a homogeneous method of determining the number of viable cells in culture through generation of a luminescent signal based on quantitation of the ATP present, an indicator of metabolically active cells [15].

### Cell surface ELISA for α-smooth muscle actin in fibrocytes

Selected human blood fibrocytes were plated at 1.5 × 10^4^ cells/well in an opaque 96 well tissue culture plate. The cells were incubated in control media (10% BSA in DMEM) overnight. The media was removed and replace with hCCL2 or transforming growth factor (TGF) β (Peprotech) in serum free media (1% BSA and ITS in DMEM). The cells were incubated for 96 h at 37°C in a humidified chamber. α-Smooth muscle actin (SMA) expression was evaluated on 96 well plates (cell surface ELISA) as previously reported [16-18]. Briefly, the cells were fixed by adding 100 μl of cold 100% methanol. The methanol was removed after 10 min and the wells were washed three times with 100 μl of PBS plus 0.5% Tween-20 (Wash buffer). The wells were then blocked with 3% H_2_O_2_ for 3 min before being washed three times with 100 μl of wash buffer. The cells were blocked a second time using DMEM containing 2% FCS for 20 min before being washed three times with 100 μl of wash buffer. The primary antibody α-smooth muscle actin (Sigma Aldrich) was added to the cells and incubated for 60 min at room temperature. The cells were washed with 100 μl of wash buffer and incubated with secondary antibody (Anti-mouse conjugated with horseradish peroxidase; Sigma Aldrich) for 60 min at room temperature. The secondary antibody was then removed and the wells were washed three times. A total of 100 μl of 2,2"-azino-bis[3-ethylbenzothiazoline-6-sulfonate] (ABTS, Sigma Aldrich) was added into the wells and incubated for 60 min at room temperature. The reaction was stopped by adding 100 μl of 1% SDS into each well. The optical density of the wells was analyzed using a plate reader at wavelength of 405 nm.

### Chemotactic migration assay

The blood fibrocytes were assayed in a 300 μl, 96-well microplate ChemoTx system using 5 μm PVP-free polycarbonate filters (Neuroprobe Inc., Gaithersburg, MD, USA) [19]. Chemotactic stimuli was CCL2 (Peprotech) diluted to different concentrations (n = 4-5 per sample condition) in 2% BSA in DMEM and added in the bottom wells of the chamber. 2% BSA in DMEM alone was added in the bottom of the chamber as a negative control (random chemotaxis). Cells were suspended in 60 μl of 2% BSA in DMEM (30 000 cells/well) and added on the top of the chamber Cells were allowed to migrate overnight at 37°C in a 5% CO_2_ incubator. After migration, cells were wiped from the top filter and the plate was centrifuged at 500 *g*. The cells were resuspended in 100 μl and cells migrating across the membrane into the lower chamber were determined using the Cell Titer-Glo Luminescence Cell Viability Assay. The chemotaxis index of hCCL2 was calculated by comparing the number of cells that migrated towards hCCL2 in comparison to the number of cells that spontaneously migrated into the bottom chamber.

### Statistical analysis

Differences between multiple groups were analyzed by analysis of variance (ANOVA) using least significant difference method. A *P *value < 0.05 was considered significant.

## Results

We assessed the phenotype and *in vitro *function of fibrocytes, or bone-marrow-derived collagen-producing cells, isolated from human and murine blood. Therefore in order to be able to directly compare the phenotype and function of human with mouse fibrocytes, we used identical isolation techniques.

### Blood fibrocytes express CCR2

**Figure 1:**
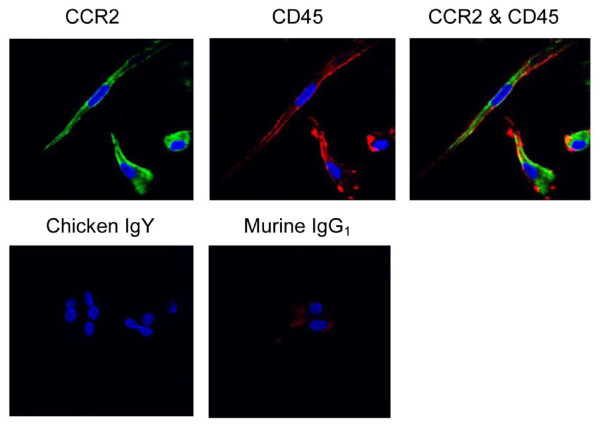
**Positive chemokine (C-C motif) receptor 2 (CCR2) expression in human blood fibrocytes by confocal microscopy**. Fibrocytes after primary passage were costained with CCR2 and CD45 or isotype controls (chicken IgY and murine IgG_1_). Nuclei were stained with 4',6-diamidino-2-phenylindole (DAPI; blue). Cells were examined at 60 × magnification. Representative images from three donors.

**Figure 2:**
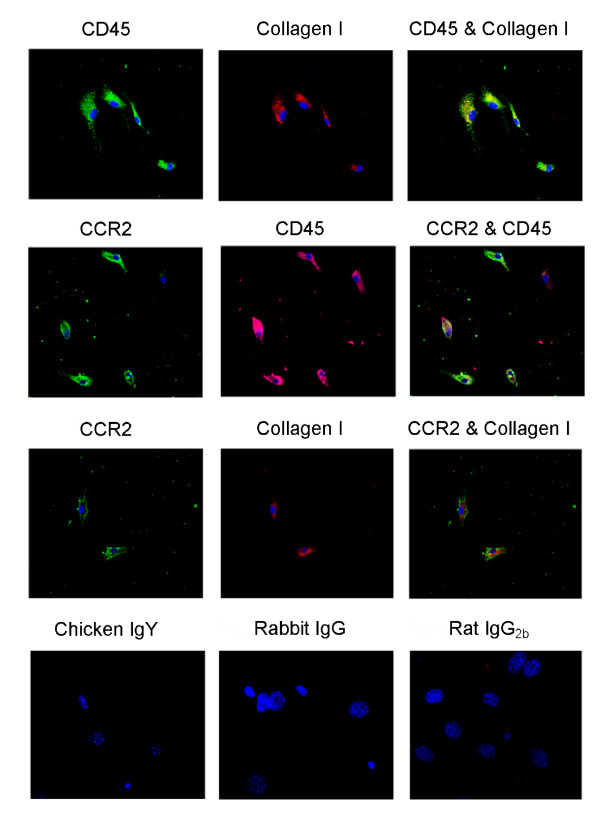
**Positive chemokine (C-C motif) receptor 2 (CCR2) expression in murine blood fibrocytes by confocal microscopy**. Fibrocyte cultures were costained with CD45 and collagen I **(A)**, CCR2 and CD45 **(B)**, or CCR2 and collagen I **(C)**. Fibrocytes were stained with isotype controls chicken IgY, rabbit IgG, and rat IgG_2b_**(D)**. Nuclei were stained with 4',6-diamidino-2-phenylindole (DAPI; blue). Cells were examined at 40 × magnification. Representative images from blood pooled from n = 30 mice.

A key morphological characteristic of fibrocytes is the irregular star or spindle shape [2,9,20]. Under our culture conditions, we observed cells from human and murine blood exhibiting this morphology (Figures 1 and 2). In the human cell system, using confocal microscopy, we detected CCR2 expression being coexpressed in the elongated CD45^+^ cells (Figure 1). In the mouse blood derived cells, the majority of cells were CD45^+^ collagen I^+^ (Figure 2). Here CCR2 expression was colocalized with both CD45^+^ and collagen I^+^ (Figure 2).

**Figure 3:**
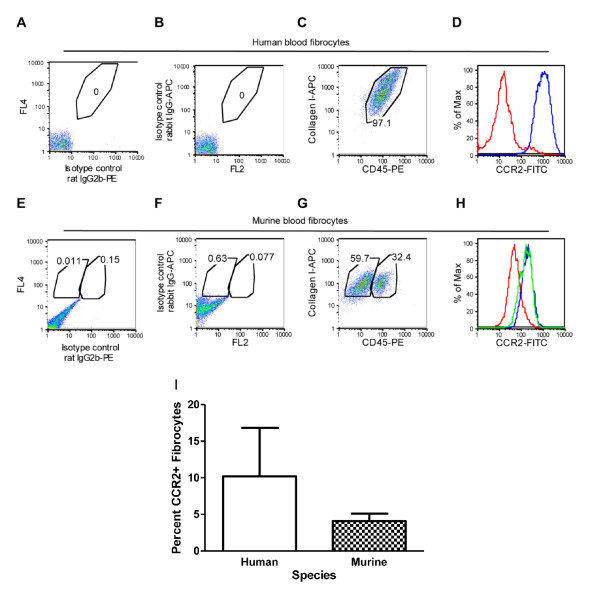
**Positive chemokine (C-C motif) receptor 2 (CCR2) expression in human and murine blood fibrocytes by flow cytometry**. Representative flow cytometric analysis of fibrocytes stained after primary passage with collagen I, CD45 and CCR2 or isotype controls. **(C) **Collagen I and CD45 staining of human fibrocytes with gate parameters calculated based in isotype controls **(A and B)**. **(D) **Representative histogram of CCR2 expression (blue) in human fibrocyte population compared to IgY isotype control (red). **(G) **Collagen I and CD45 staining of murine fibrocytes with gate parameters calculated based in isotype controls **(E and F)**. **(H) **Representative histogram of CCR2 expression (blue) and collagen I (green) compared to relevant isotype control (red). **(I) **Quantitative analysis of percentage of CCR2+ cells in fibrocyte population. Data are expressed as the mean ± SEM of n = 4 human donors and n = 30 mice.

To quantify the expression of CCR2 on human and murine cultured fibrocytes, freshly isolated peripheral blood mononuclear cells were triple stained with CD45, collagen I and CCR2 and enumerated by flow cytometry. Approximately 97% of the human blood fibrocytes were CD45 and collagen I positive (Figure 3C). Of this population, the cells were strongly positive for CCR2 (Mean fluorescence intensity (MFI) = 1221) compared to the IgY isotype control (MFI = 41) (Figure 3D). In mice, approximately 92% of the cultured blood cells stained strongly for collagen I and 32% of this population also expressed CD45 (Figure 3G). A time-dependent loss of CD45 in human fibrocyte culture has been observed previously [9]. Therefore, reduced CD45 expression in the murine versus human fibrocytes in this study may have been due to the extended time required for mouse fibrocyte culture. We observed that murine blood fibrocytes (MFI = 230) and the CD45^-^ collagen I^+^ (MFI = 186) were strongly positive for CCR2 compared to the IgY isotype control (MFI = 63) (Figure 3H). When comparing multiple human donors and mouse blood samples, approximately 10% of human fibrocytes and 4% of mouse fibrocytes are CCR2 positive (Figure 3I).

### CCL2 increases blood fibrocyte proliferation

**Figure 4:**
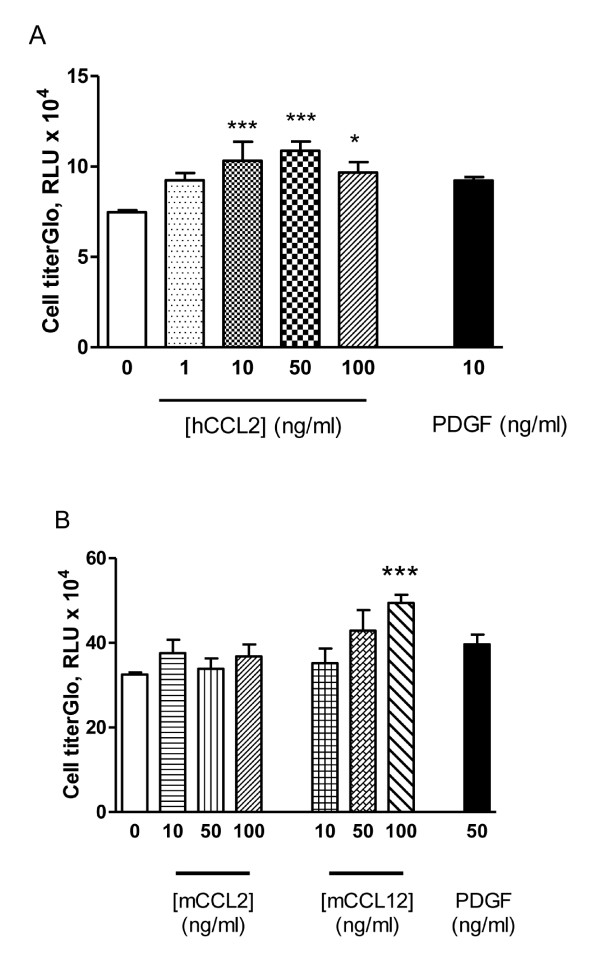
**Chemokine (C-C motif) receptor 2 (CCR2) ligand promotes proliferation of human and murine blood fibrocytes**. Human blood fibrocytes **(A) **were stimulated with human chemokine (C-C motif) ligand 2 (hCCL2) or platelet-derived growth factor (PDGF)-AB and murine blood fibrocytes (pooled blood from n = 10 mice) **(B) **were stimulated with mCCL2, mCCL12 or PDGF-AB. Cells were grown for 4 days before proliferative activity was measured using CellTiter-Glo Luminescent Cell viability. *P < 0.05, significantly different compared with untreated fibrocytes. ***P < 0.005, significantly different compared with untreated fibrocytes. Data are expressed as the mean ± SEM (n = 3 replicates per treatment group) (representative of three separate experiments).

Fibroblast proliferation is a key hallmark of IPF [21]. We next examined whether fibrocyte proliferative capacity could be increased by stimulation with CCR2 ligands. Here, we were able to further demonstrate that blood fibrocytes have functional CCR2, as exogenous CCR2 ligands significantly enhanced proliferation *in vitro *(Figure 4). In the human blood fibrocytes, 10 ng/ml of hCCL2 induced a significant increase in fibrocyte proliferation, with 50 ng/ml of hCCL2 inducing the greatest effect (*P *< 0.005), with a 45% increase on cell proliferation (Figure 4A). In the CCR2 ligand assessments, murine fibrocytes were responsive to mCCL12 with both 50 and 100 ng/ml inducing proliferation (Figure 4B). However, mCCL2 did not mediate a significant induction of fibrocyte proliferation at any of the concentrations tested (Figure 4B). In both the human and murine fibrocytes, PDGF, a fibroblast mitogen [22] also produced a modest increase in fibrocyte proliferation by approximately 20% (*P *> 0.1) (Figure 4), but was not as potent as exogenous CCR2 ligands.

### CCL2 enhanced blood fibrocyte differentiation into myofibroblast phenotype

**Figure 5:**
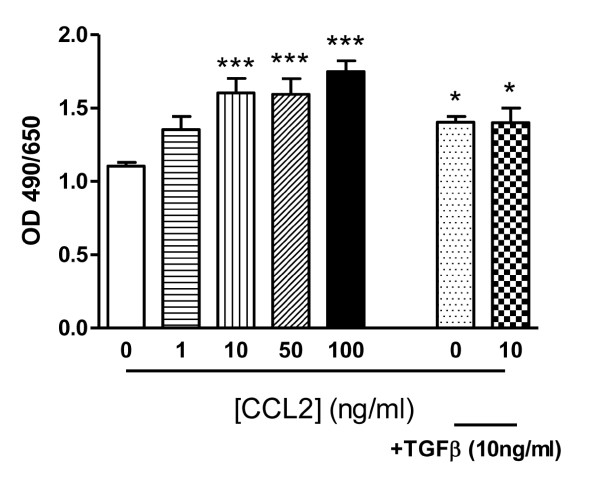
**Chemokine (C-C motif) receptor 2 (CCR2) ligand induces differentiation of human blood fibrocytes**. Human blood fibrocytes were stimulated with human chemokine (C-C motif) ligand 2 (hCCL2) or transforming growth factor (TGF)β and grown for 4 days before α-smooth muscle actin levels were analyzed. The optical density (OD) reading is the level of α-smooth muscle actin, which relates to the amount of fibrocyte differentiation into myofibroblasts. *P < 0.05, significantly different compared with untreated fibrocytes. ***P < 0.005, significantly different compared with untreated fibrocytes. Data are expressed as the mean ± SEM (n = 3 replicates per treatment group) (representative from three separate experiments).

The fibrotic lesions observed in the lungs of IPF patients contain α-SMA^+^ myofibroblasts [21]. Fibrocytes are hypothesized to migrate to the lung and differentiate into cell types reminiscent of fibroblasts or α-SMA^+^ myofibroblasts. To determine if CCR2 has a role in this hallmark feature of fibrosis, we studied whether human fibrocytes could differentiate into myofibroblast-like cells in response to TGFβ or hCCL2. TGFβ is an important fibrogenic and growth-regulating cytokine involved in tissue remodeling and is a potent inducer of fibroblast to myofibroblast differentiation, as well as monocyte to fibrocyte differentiation [1,3,4,23,24]. In our study, culturing human blood derived fibrocytes in serum-free media results in the expression of α-SMA at the protein level (Figure 5). However, administration of exogenous TGFβ increased α-SMA expression by 24% in human blood fibrocytes. hCCL2 also increased α-SMA protein expression, with 100 ng/ml of hCCL2 mediating the greatest induction of α-SMA (54%, *P *< 0.001, Figure 5).

### CCL2 promotes fibrocyte migration

**Figure 6:**
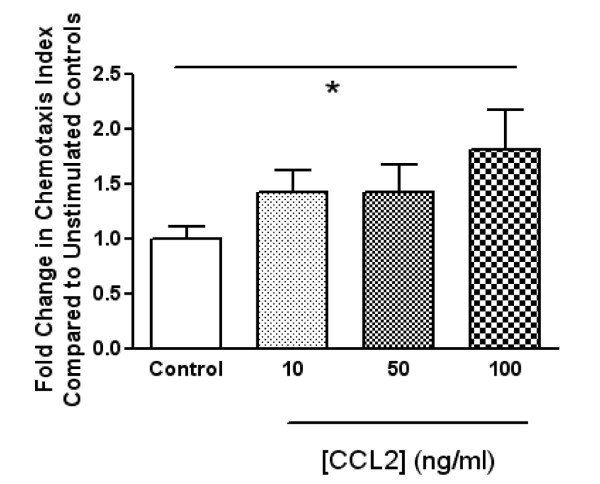
**Chemokine (C-C motif) ligand 2 (CCL2) induced chemotactic response in human blood fibrocytes**. Human blood fibrocytes were subjected to chemotaxis in response to the indicated hCCL2 concentrations overnight. *P < 0.05, significantly different compared with untreated fibrocytes. Data are expressed as the mean ± SEM (n = 3 healthy humans performed in quadruplicate per treatment group).

As fibrocytes are circulating bone-marrow-derived cells, we next assessed the ability of these cells to undergo chemotactic migration in response to hCCL2. Human isolated fibrocytes were added to the bottom chamber of a chemotaxis plate and increasing concentrations of hCCL2 were added to the top chamber. Only 100 ng/ml of hCCL2 was able to induce a significant migratory response in these cells (*P *< 0.05) (Figure 6). There was a non-significant trend with the lower concentrations of hCCL2.

## Discussion

Circulating fibrocytes have been described to be one of the potential sources of increased extracellular matrix and myofibroblasts that are hallmarks of fibrotic pathologies [8,25]. Fibrocytes derived from the bone marrow migrate to sites of tissue injury and may enhance fibrosis directly through the production of collagen and profibrotic factors [14,25,26]. Inhibition of fibrocyte recruitment and/or activation could thus allow distinct therapeutic paths to treat a range of fibrotic diseases. We have shown that both human and murine fibrocytes express CCR2 and stimulation of this receptor with relevant chemokine ligands results in profibrotic responses including differentiation and proliferation. In experimental models of lung fibrosis, blockade of CCR2 ligands results in a reduction in collagen deposition [6,7]. Therefore this study directly confirms CCR2 expression and function between mouse cells and human cells which may directly translate into human disease.

Human peripheral blood fibrocytes have been shown to express a variety of chemokine receptors including CCR7 and CXCR4 [8,25]. Murine models have implicated a role for fibrocytes in contributing to the pathogenic remodeling during pulmonary fibrosis [7,14,25]. However, translating function and effect on chemokine neutralization, to human clinical disease has yet to be demonstrated. In this study, we showed that CCR2 is expressed on human and murine blood fibrocytes. A previous study in using fibrocytes isolated from fibrotic lung tissues revealed the murine CCR2 receptor to be highly expressed and to mediate migration *ex vivo *[6]. Human and murine peripheral blood fibrocytes have also been shown to express CCR2 [27,28]. We have extended these studies by assessing other chemokine-associated functions. Moreover, importantly we have shown a commonality between the murine and human system, in that both human and murine cultured fibrocytes express functional CCR2. A natural extension of these studies would be to assess and compare CCR2 on fibrocytes isolated from patients with IPF with those isolated during murine models of lung fibrosis.

To investigate the functional responses to CCR2 activation, we analyzed the effects of CCR2 ligands on various activation responses in cultured blood-derived fibrocytes. We focused on processes associated with fibrosis such as differentiation to myofibroblasts, proliferation and migration. Fibrocytes are extremely adherent cells when isolated and cultured *in vitro*. However, CCL2 promoted the chemotactic migration of fibrocytes. Studies have demonstrated increased CCL2 in the circulation [29] and in the lungs [30,31] of IPF patients and there is evidence for a possible concentration gradient that may enable chemoattraction activity of CCL2 [31]. Therefore, this chemokine axis may be central to the observed increase in the number of fibrocytes seen in the blood [13] and lungs [32] of IPF patients.

Previous reports have linked IL-1β and tumor necrosis factor α, to enhancing fibrogenic responses *in vivo*, and demonstrated an increase in the secretion of chemokines (CCL2 and CXCL8) and hematopoietic growth factors (IL-6 and M-CSF) in human peripheral blood fibrocytes [3]. We observed no significant CCL2-induced secretion of cytokines by human and murine peripheral blood fibrocytes (data not shown). Key hallmarks of active fibrotic lesions are the presence of myofibroblasts and a proliferation of collagen producing cells [33-35]. TGFβ and endothelin-1 (ET-1) can promote fibrocyte differentiation into myofibroblasts resulting in a cell population producing increased collagen and having contractile properties [1,4]. We evaluated whether human blood fibrocytes could differentiate into myofibroblast-like cells under the influence of CCL2. Here, we show that CCL2 promotes myofibroblast differentiation, as detected by increased α-SMA expression. Recent studies in human blood fibrocytes have shown that a variety of mediators including TGFβ and cysteinyl leukotrienes modulate fibrocyte proliferation and interestingly TGFβ induced effects on IPF fibroblasts that are partly mediated via CCL2 [4,36].

Here, we demonstrate that exogenous hCCL2 and mCCL12 lead to increased blood fibrocyte proliferation in human and murine cells respectively. Interestingly, mCCL2 did not induce mouse fibrocyte proliferation. Both mouse chemokines have been shown to bind to and signal through CCR2 with similar potencies [22]. However, it is has been proposed that mCCL12 may utilize another receptor in addition to CCR2 [22]. In the human blood fibrocyte studies, human cells responded to low levels of hCCL2 stimulation. There is no human homologue of mCCL12; therefore this data suggests that human CCL2 may be more like murine CCL12 with respect to fibrocyte function.

## Conclusions

In summary, the current study, using an optimized fibrocyte isolation technique from both mouse and human blood, has demonstrated the presence of functional CCR2 on a subpopulation of human and murine blood fibrocyte cells. Signaling through ligand-CCR2 interactions in both human and murine blood fibrocytes induced fibrocyte proliferation, differentiation of fibrocytes into myofibroblasts, and a chemotactic response. Comparable CCR2 expression and biological activity of CCR2-CCL2/CCL12 in both murine and human fibrocytes was detected. Preclinical murine models of lung fibrosis have demonstrated potent therapeutic activity mediated by inhibiting this chemokine axis [6,7,10]. Additionally, fibroblasts from the lungs of IPF patients are hyper-responsive to CCL2, whereas fibroblasts isolated from non-fibrotic lesions do not respond to CCL2 [10]. Taken together, this highlights the potential for an anti-CCR2/CCL2 approach at inhibiting human lung fibrosis through attenuating fibroblast activity directly as well as reducing downstream fibrocyte profibrotic activities.

## Competing interests

Centocor R&D sponsored this study. All authors were employed by Centocor R&D during this study.

## Authors' contributions

JEE and LAM conceived the study, participated in design and coordination, and drafted and wrote the final manuscript. SH participated in conducting the studies. MAR, JK and AMD participated in design and coordination. All authors read and approved the final manuscript.
